# Operating hurts: a study of EAES surgeons

**DOI:** 10.1007/s00464-018-6574-5

**Published:** 2018-11-19

**Authors:** Antonia C. Wells, Magnus Kjellman, Simon J. F. Harper, Mikael Forsman, M. Susan Hallbeck

**Affiliations:** 1 Department of Surgery, Cambridge University Hospitals NHS Foundation Trust, Cambridge, UK; 2 Department of Molecular Medicine and Surgery, Karolinska Institutet, Stockholm, Sweden; 3 Institute of Environmental Medicine, Karolinska Institutet, Stockholm, Sweden; 4 Division of Ergonomics, KTH Royal Institute of Technology, Stockholm, Sweden; 5 Health Sciences Research, Mayo Clinic, 200 First St SW, Rochester, MN 55905 USA

**Keywords:** Survey, Musculoskeletal pain, Burnout, Operating theatre, Surgical ergonomics, Endoscopy

## Abstract

**Background:**

Work-related pain and discomfort experienced by surgeons is widely reported in the literature. A survey was, therefore, conducted to explore this issue among members of the European Association for Endoscopic Surgery (EAES).

**Methods:**

The survey was emailed to 2980 EAES members in 2017 enquiring about their working practice, musculoskeletal (MSK) pain and burnout.

**Results:**

A total of 569 (19%) surgeons responded, of whom 556 were practicing surgeons; 86% were consultants, 84% were male, and 94% were right-handed. Respondents operated on average 3.3 days/week with 27% of their procedures lasting longer than 3 h. The 386 endoscopists surveyed reported performing an average of 5.3 procedures/day with 83% performing endoscopy at least once per week. Over half of practicing surgeons (62%) reported their worst pain score was 3 or higher (10-point scale) in the past 7 working days, encompassing 71% of their open, 72% laparoscopic, 48% robot-assisted cases and 52% of their endoscopies. Of the 120 surgeons who had ever sought medical help for aches, pain or discomfort, 38% were currently in pain and 16% had considered leaving surgery due to their MSK pain, 26% had reported work-related pain to their employer, 26% had been on short-term disability during their career and 4% long-term disability due to MSK disorders. A significant proportion of the respondents (49%) felt their physical discomfort would influence the ability to perform or assist with surgical procedures in the future. These surgeons reported significantly lower satisfaction from their work (*p* = 0.024), higher burnout (*p* = 0.005) and significantly higher callousness toward people (*p* < 0.001) than those not fearing loss of career longevity.

**Conclusion:**

The results show that MSK pain is prevalent amongst EAES members. Nearly half the respondents had career longevity fears from pain/discomfort which, in turn, correlated with more prevalent feelings of burnout. More emphasis should be placed on the aetiology, prevention and management of musculoskeletal pain in the surgical workforce.

Performing surgery imposes physical challenges with potentially long-lasting consequences for practitioners. Surgeons frequently operate in relatively static positions with sustained, often awkward postures, resulting in stiffness and pain that often persist outside of the operating room [1, 2]. Important publications such as “While patients benefit, surgeons suffer” [2] and “The operation room as a hostile environment for surgeons” have highlighted the detrimental impact of poor ergonomics on the surgical workforce [3–5].

When surveyed, the proportion of surgeons reporting pain during or after surgery is consistently high, ranging from 60% [3, 4] to nearly 90% [1, 2, 6–9]. In keeping with this, Alleblas, et al. found in a systematic review that the prevalence of musculoskeletal (MSK) morbidity among surgeons was 74% and even if all non-responders were assumed to have never experienced MSDs, the adjusted prevalence was 22% [10]. Moreover, the incidence of surgeons experiencing MSK discomfort, pain and dysfunction appears to be increasing [11]. This is likely in part due to the increase in procedures performed using minimally invasive surgery. Very few engineering controls exist in the operating room (OR) to improve posture; indeed OR equipment and environment designs often induce poor working posture [12–14]. This is particularly pertinent to those performing minimally invasive surgery (MIS) who experience a range of work-related injuries that can affect nerves in the arm and hand, cause rotator cuff injuries, and damage cervical joints [15]. Over 9% of surveyed MIS surgeons have been forced to stop practicing due to disability or MSK-related pain [16, 17]. Surgeons may choose to perform open surgeries rather than MIS techniques because of MIS-related pain, which may in turn increase patient recovery time [18].

Surgical team members, including trainees, also experience high levels of burnout [19, 20]. The causes of burnout are complex and comprise both mental and physical stressors, including work-related MSK morbidity. This issue highlights a potentially serious problem, in that, while surgical productivity falls due to work-related injury, demand from patients for complex minimally invasive procedures continues to grow [21–23].

The aim of this study was to survey EAES members to identify the prevalence of MSK pain/ discomfort and identify any associations with surgical practice, working conditions, burnout and quality of life.

## Materials and methods

After Institutional Review Board Approval (17-003472), a survey was emailed to all members of the European Association of Endoscopic Surgery (EAES) between May 19 and July 17, 2017. An anonymous link to a Qualtrics (Qualtrics, Provo, UT) survey was emailed from Mayo Clinic’s Survey Research Center to 2980 active EAES members about their working practice, MSK pain, discomfort and burnout. The survey was based on Park et al. [9], previous questionnaires by Berguer [5], Park et al. [2], Plerhoples et al. [4], the Brief Pain Inventory (BPI) measuring quality of life [24] using the Borg CR-10 scales [25], the Nordic Musculoskeletal Questionnaire (NMSQ) [26] and burnout [27]. The Qualtrics (v. 2017; Qualtrics, Provo, UT) survey had a maximum of 43 items to complete, but in practice, it included a lower number, due to branching logic for some questions.

The first question asked was whether they were currently performing surgery; if not, were they retired, had to stop due to MSK discomfort, pain or injury, change of career or other. Those members not currently performing surgery answered 3 burnout questions [27], and demographics (age, gender, BMI and dominant hand).

Those currently performing surgery were asked about their primary surgical division. Questions using the 0–10 Borg’s CR-10 scale [25] to measure pain/discomfort [24] and percentage of their time spent performing Open, Laparoscopic and Robotic Surgeries and Endoscopy in the past 12 months were asked. The NMSQ [26] questionnaire for musculoskeletal symptoms was used to rate body part discomfort on a scale from 0 (none) to 10 (worst imaginable), for discomfort both related to surgery and overall for the last 7 working days. The body locations queried were neck, shoulder, upper back, lower back, right thumb, right fingers, left thumb and left fingers. They were also asked to rate their overall worst pain level during the past 7 working days.

The surgeons were asked at what point during their operative day they noticed body discomfort attributable to surgery and whether they felt that their discomfort would influence their ability to perform surgery [2]. They were asked how the discomfort impacted on their life (including sleep and relationships [24]) and what they had done to mitigate the operative pain or discomfort (adapted from the SAGES questionnaires [5, 28, 29]).

If they were still in pain, they answered questions on accessing medical help, if they had reported symptoms to their employer, what medical help they received and any time taken off work for treatment. The last portion of the survey consisted of demographic questions including type of practice setting, types of procedures performed, frequency and duration of those procedures [29].

Descriptive statistics, t-tests and Pearson product-moment correlations were performed on the data. The statistical significance level was set at *α* = 0.05.

## Results

### Demographics

Of the 2980 EAES members current in May 2017, 569 surgeons responded (19%). It is important to note that not every respondent answered every question which explains the varying total numbers presented. There were 556 practicing surgeons; 86% were consultants, 84% were male and 94% were right-handed. Their BMI averaged (sd) 25.7(3.5). The respondents were spread over the different types of practice: 32% were in a government hospital practice, 21% were in private practice and 33% were in an academic medical center/hospital practice setting. Of the 13 no longer practicing surgery, 6 had retired, 2 had to stop due to MSK discomfort/pain/injury, 2 had changed careers and 3 reported “other”. Table 1 has the age demographics of EAES members responding to the survey.

**Table 1:** Age demographics of respondent EAES members

Age range	% reporting
20–30	2.8
31–40	29.0
41–50	33.3
51–60	25.0
61–70	9.6
71+	0.3

The respondents reported operating on average(sd) 3.3(1.2) days/week with 27%(23%) of their surgeries lasting longer than 3 h. Fourteen percent used loupes when operating and 18% wore a headlight during their surgeries. Most participants ticked more than one box for surgical modality (open, laparoscopy, robotic or endoscopy).The 386 endoscopists performed an average of 5.3(3.6) procedures/day with 83% performing endoscopy at least once per week.

### Pain in the past 7 days

Overall, 87% of the respondents reported they had work-related musculoskeletal pain in the last 7 working days. 62% of practicing surgeons reported a significant pain score, i.e. a score of 3 or higher (on a 0–10 scale), in the last 7 working days. Pain scores of 1 or 2 were reported by 25% of the respondents, while 13% reported no pain.

The surgeons were also asked about their *worst* overall pain/discomfort during the previous 7 working days. The mean (sd) pain score overall was 3.8(2.7) out of 10 for their worst pain in the past 7 working days across all body parts; however, the range was 0–10.

**Fig. 1:**
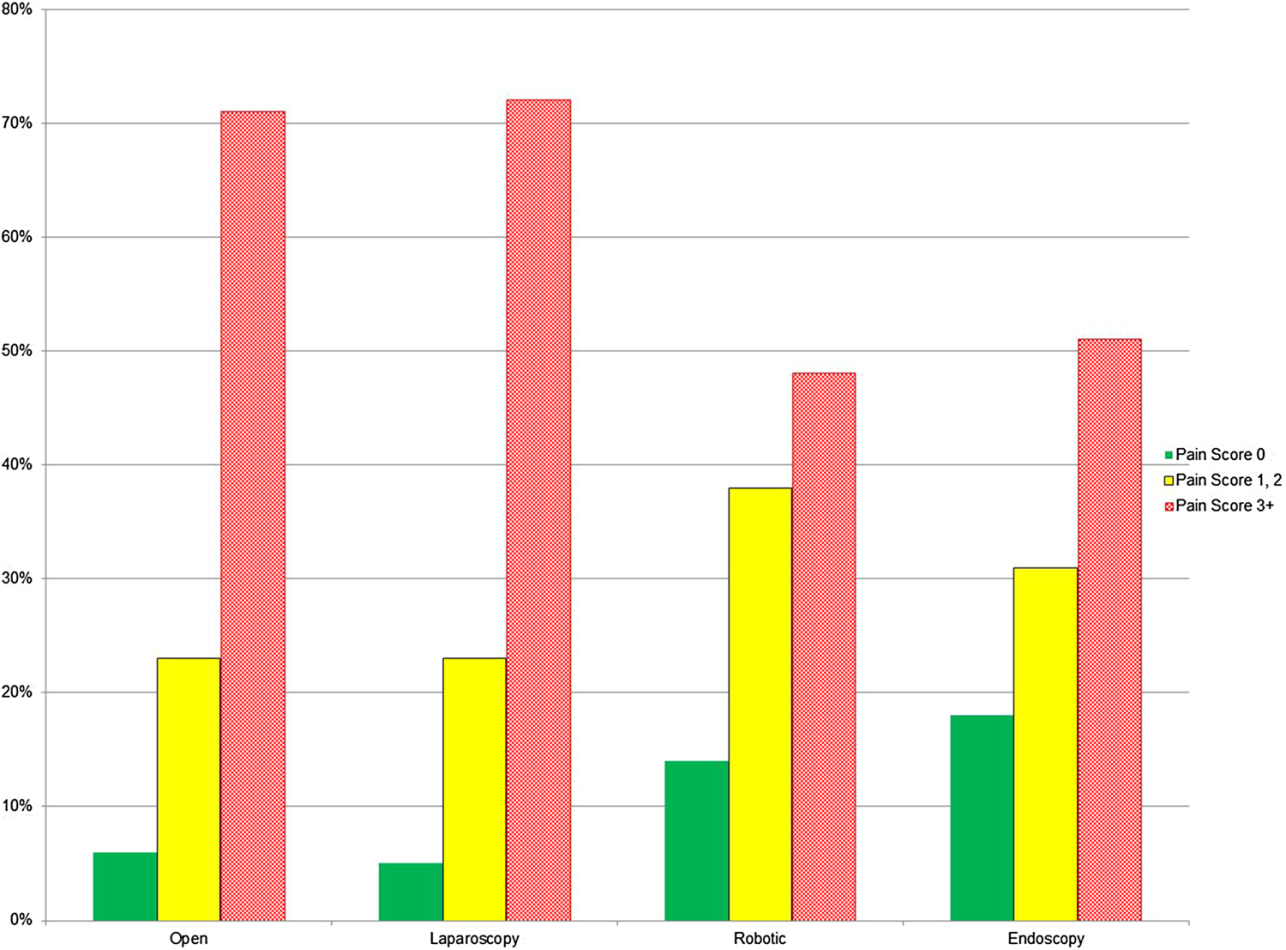
Percentage of respondents who reported pain, in the last seven working days, by pain score and body part

The percentage of those reporting pain of 3/10 or higher immediately after surgery are 52% in the neck, 46% in the shoulder, 41% in the upper back and 52% in the lower back. Surgeons reported significant pain from performing surgery in the back, neck and shoulders, in particular, as shown in Fig. 1.

### Surgical modality and pain over the past 12 months

**Fig. 2:**
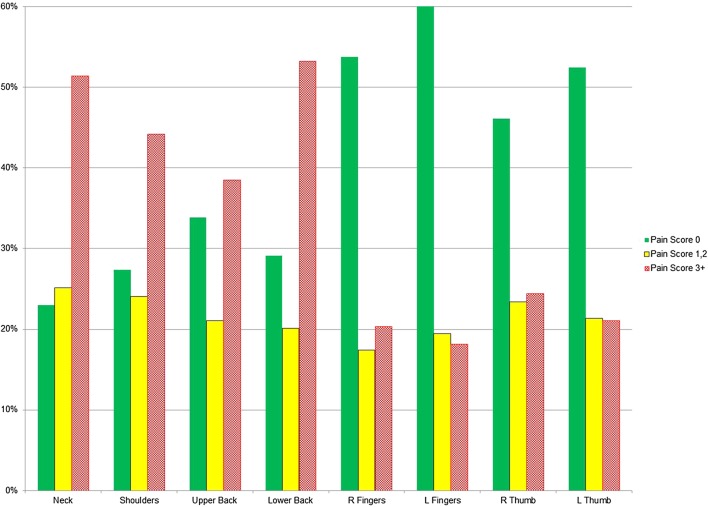
Percentage of respondents who reported pain in the last 12 months, by surgical modality

The EAES member surgeons were then asked about pain/discomfort in the last 12 months by operating modality. When examined by surgical modality, 71% of respondents reported significant pain for their open, 72% for laparoscopic, 48% for robot-assisted (RAS) cases and 51% for their endoscopies, as shown in Fig. 2.

During the last 12 months, severe pain (7 and above) was reported by 16% of open, 22% of laparoscopic, 6% of robotic and 11% of endoscopic surgeons.

### Occupational reporting and impact on quality of life

**Fig. 3:**
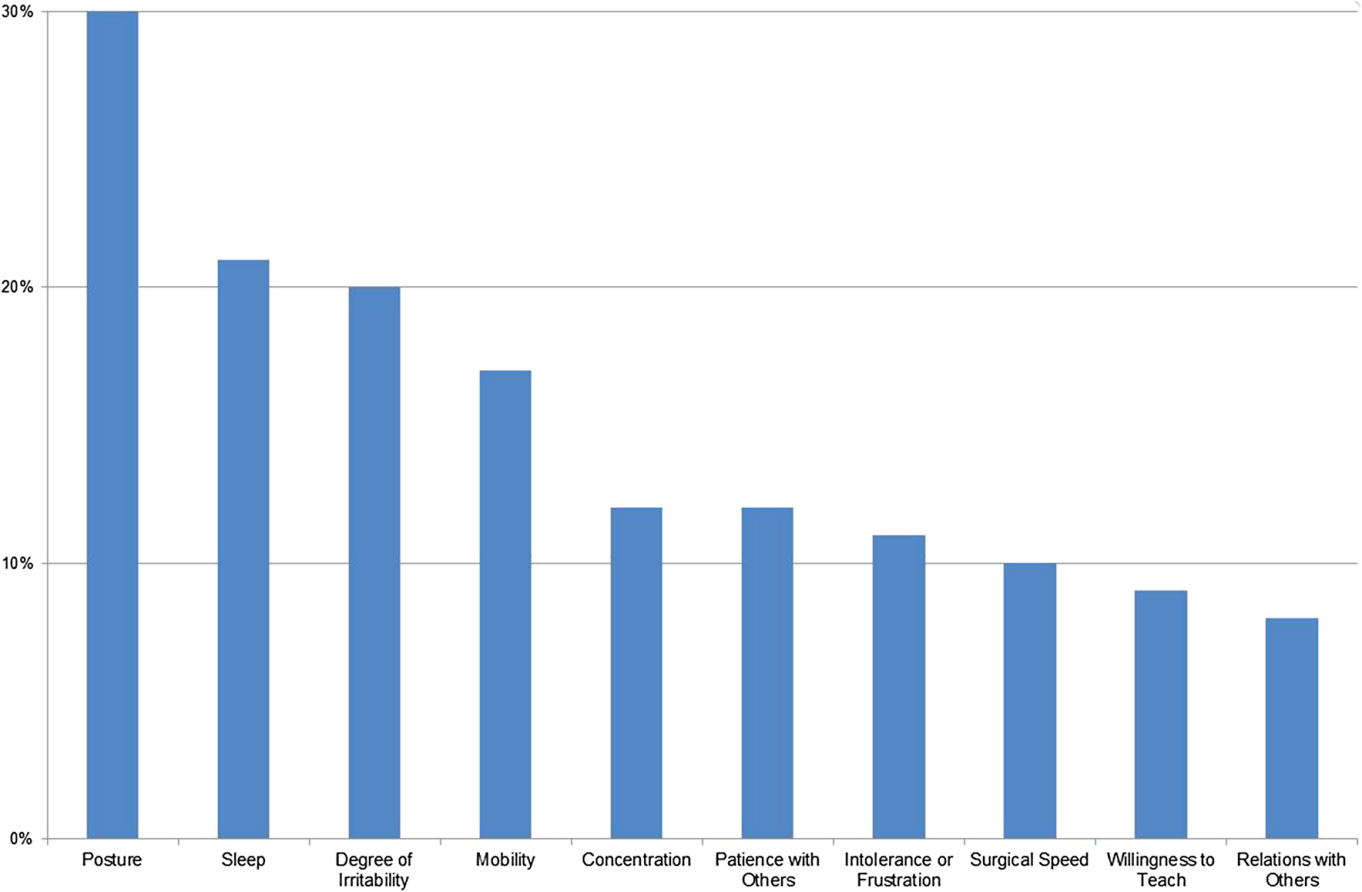
Impact of discomfort for all respondents, percentage reporting each impact

Only 26% of the respondents had reported work-related pain to their employer. Despite this, there was a large impact from their discomfort on various aspects of their quality of life (QOL) such as sleep, irritability, relations with others, etc. is shown in Fig. 3. Since respondents could tick off more than one box, it does not sum to 100%.

**Fig. 4:**
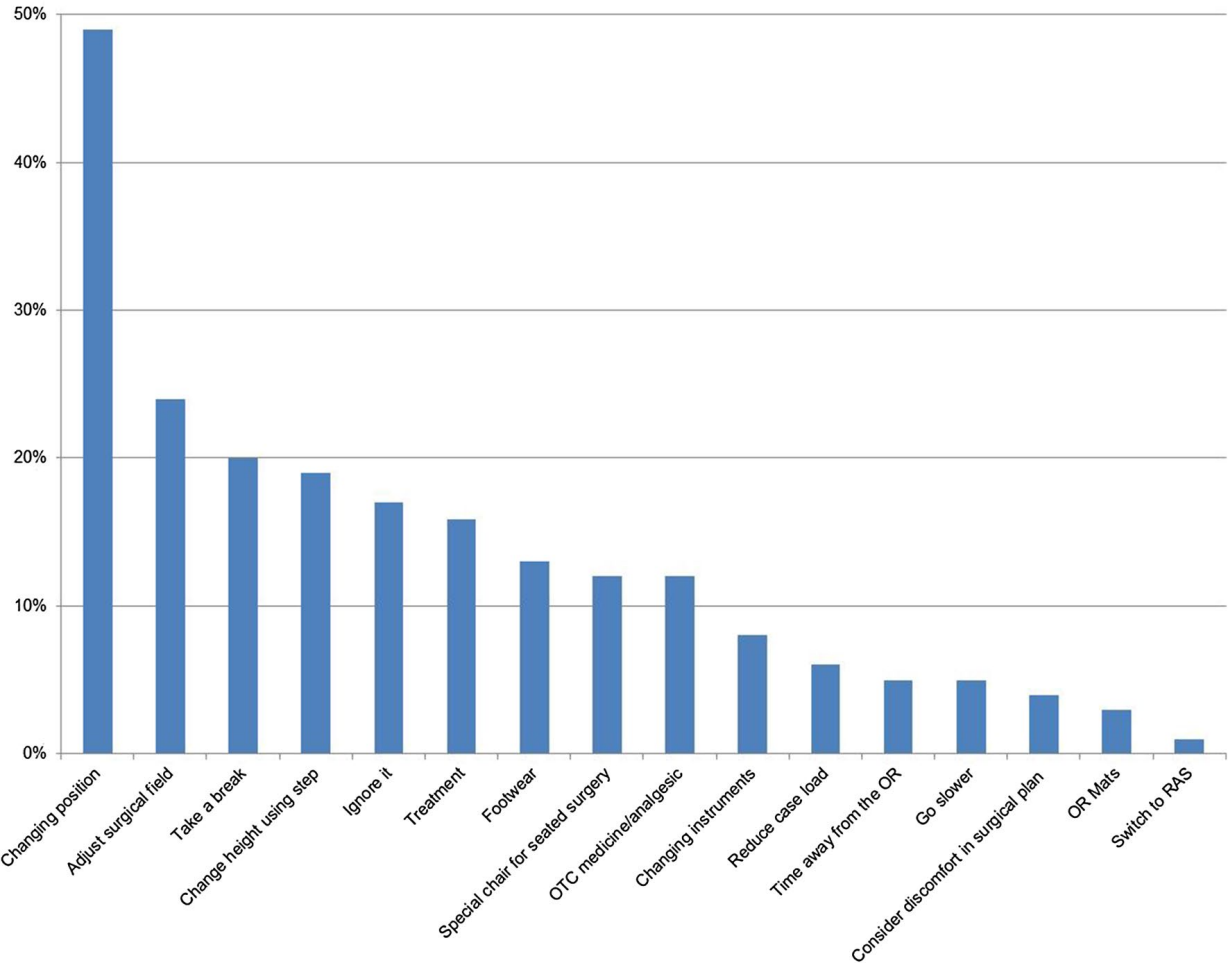
Percentage of surgeon respondents reporting physical discomfort mitigation

The surgeons were also asked how they dealt with the pain and discomfort. Most attempted minor adjustments (e.g. changing position or adjusting the surgical field), ignoring it or taking a break. A much smaller proportion of those surveyed adopted a more long-term or systematic approach such as switching surgical modality, considering the operative approach, reducing caseload or using OR mats, as shown in Fig. 4.

Of the 556 respondents, 120 surgeons, representing 22% of the overall respondents, had sought professional medical help for their pain or discomfort. The type of medical help or treatment employed by this subgroup of surgeons is shown in Table 2. The most common treatment is prescription medication.

**Table 2:** Type of treatment for those surgeons who sought professional medical help

Type of medical treatment sought	*n* = 120	%
Prescription medication	68	57
Massage therapy	63	53
Physiotherapy	58	48
Diagnostic studies	56	47
Referral to a specialist	42	35
Brace or support device	14	12
Surgery	12	10
Chiropractic	11	9
Acupuncture	10	8
Cervical traction	8	7

Of the 120 respondents who had sought medical help for their pain or discomfort, 38% were currently in pain and 16% had considered leaving surgery due to their MSK pain. Of those surgeons who had work-related pain, 19% had missed work at some time, 15% had missed up to 1 week for medical treatment or rehabilitation, 6% had missed 2–4 weeks and 1% had missed more than 4 weeks of work. 26% had been absent from work on short-term disability during their career with 4% on long-term disability due to MSK disorders. Only 15% reported having had an ergonomic evaluation of their workplace.

The duration of surgery can impact discomfort and pain. 20% of all respondents had pain or discomfort within the first hour of performing surgery. Cumulatively, 39% had discomfort before the 2-h mark, 66% before 4 h, and 85% before 6 h of surgery were completed, only 3% never felt discomfort while performing surgery.

Overall, 27% of the respondents’ surgeries last 3 or more hours, leading to the conclusion that many surgeons are in pain during surgery.

### Burnout

Overall, 12% of all the respondents were at a high risk of burnout, 7% of all the respondents were at a high risk for de-personalisation (callousness) and 4% for high dissatisfaction, using West et al. definition for low and high risk [27]. Conversely, 48% were at low risk for burnout, 65% were at low risk for de-personalisation and 83% reported high satisfaction with their work life.

Forty-nine percent of reporting respondents felt that their work-related physical discomfort would influence their ability to perform or assist with surgical procedures in the future. These surgeons reported significantly lower satisfaction from their work (*p* = 0.024), significantly higher burnout (*p* = 0.005) and significantly higher callousness toward people (*p* < 0.001) than those not fearing loss of career longevity. Surgeons who had been on short-term disability reported significantly lower satisfaction from their work (*p* = 0.034) than those who had not been on disability. Surgeons who rated their work-related discomfort had impacted their sleep, their patience with others and their intolerance or frustration also reported higher feelings of burn out, with a median of 4/10 compared to 2/10 for surgeons not reporting work-related discomfort. Similarly, those with higher burnout (4/10 versus 2/10) took over-the-counter medicine/analgesic and had massage therapy as resilience measures.

There was a significant correlation (*p* < 0.0.5) between higher satisfaction and lower pain scores for overall pain from endoscopy, open, laparoscopic and robotic surgeries as well as by body part, including neck, upper and lower back, right and left thumb and fingers and that surgeon’s worst pain in the last 7 days. Significant correlation between higher satisfaction and surgeries shorter than 3 h was found, as was between higher satisfaction and performing more surgeries per week. Significant correlations were found between higher reported burnout ratings and increased overall pain from endoscopy, open, laparoscopic and robotic surgeries. There were significant correlations between higher burnout and pain by body part for the neck, upper and lower back, and also the surgeon’s worst pain in the last 7 days. The results from the correlations between de-personalisation and surgical modality, body part and worst pain were significant and the correlation coefficients were similar to those for burnout. While all these correlations were significant, the correlation coefficients for each pair were all lower than 0.707 which is where one variable predicts 50% of the variation in the other variable.

## Discussion

While there have been numerous publications demonstrating the significant burden of work-related MSK morbidity among surgeons, it remains a poorly reported problem within medical institutions. This issue can have a significant impact on the lives and careers of individual surgeons and be detrimental to patient care and surgical productivity [15–23]. The aim of this study was to gain greater insight into MSK morbidity in the surgical workforce using a comprehensive survey of EAES members. The results of this study indicate that MSK pain is a major issue for significant numbers of EAES surgeons, and by logical extension, is likely to reflect wider surgical experience. Indeed, MSK pain during surgery appears to affect the vast majority of surgeons, with only 3% of those surveyed reporting pain-free operating.

Open and laparoscopic procedures are associated with more significant pain than robotic and endoscopic approaches, although for all modalities the prevalence of significant pain during the last 12 months was above 50%. The reality is that most surgeons perform a combination of surgical procedures (other than robotic surgery, which currently remains a low frequency for this population) and are, therefore, exposed to the MSK stressors associated with a variety of techniques. Unsurprisingly, longer operations are associated with more significant pain and by 6 h into a surgical procedure, 97% of surgeons experience pain. Perhaps more importantly though, 40% experience pain after just 2 h, indicating that many surgeons will experience problems in a significant proportion of their cases.

The overall mean pain score of 3.8 (sd 2.7) suggests that the symptoms being reported are not ‘minor twinges’ but are in fact having a significant impact on surgeon well-being. In keeping with this, surgeon discomfort appears to be related to lower job satisfaction, de-personalisation, burnout and ability to continue practicing. Indeed, nearly half the respondents had career longevity fears and of the 120 surgeons who had ever sought medical help, a third had been on short or long-term disability leave. Importantly, only 26% of those in pain had reported this to their employer and interventions adopted to manage operative MSK pain were predominantly short-term and temporary.

This level of pain prevalence and career longevity fear are likely high in comparison to other professional fields. With respect to the pain scores, about 52% of the respondents reported they had work-related musculoskeletal pain in the neck in the last 7 working days. This is as high as reported for women in repetitive/constrained industrial work [30]; however, in that study, the figure included all pain (not just self-rated as work-related). Among women with varied/mobile work the corresponding percentage was 34%, for men these percentages were 32% (repetitive/constrained industrial work) and 29% (varied/mobile work). In addition, when comparing the 12-month prevalence of pain with other health-care workers, from a review study; [31] including dentists, for which 7 of 12 studies found neck pain prevalence above 50%, the present percentages, especially for open surgery and laparoscopy (both above 70% prevalence of work-related pain), are indeed high.

Finally, concerning fear about career longevity, about half of the respondents felt that their work-related physical discomfort would influence their ability to work with surgery in the future. A study by Lundin et al. [32] included a random sample of the working population who rated their ‘own prognosis of their work ability 2 years from now’, on a 3-level-scale where 12% rated the highest level of career longevity fear. Although the questions in that and in the present study were slightly different, the comparison indicates that the career longevity fear is unusually high among the responding surgeons.

One of the key advantages of this and similar studies is that it allows surgeons to respond in detail in regard to their MSK pain with complete anonymity. This facilitated more honest reporting as many surgeons may be reluctant to admit to these issues for fear of it impacting negatively on their career. The main disadvantage of a study of this type is the concern over reporting bias, in particular, that a higher proportion of ‘responders’ have MSK pain to report and those without pain are less likely to take an interest in such a study. In order to minimise this effect, the survey was emailed during the 2017 EAES congress where potentially more surgeons had the time and opportunity to complete the study. Most studies in this area, however, concur that MSK pain amongst surgeons is much more likely to be under-reported [10], with many surgeons assuming it is ‘just part of the job’ [9, 33]. Another key challenge in a study of this type is separating general MSK symptoms from those directly related to work. Similarly, the direction of causality can be impossible to completely resolve. For example, do MSK problems lead to increase burnout or does burnout exacerbate the impact of MSK symptoms? The likely answer is that probably both are true. This area of research is complex, multifaceted and subjective and the results on the whole provide an impression of the problem rather than concrete answers. We believe these results will help create the parameter to craft concrete interventions to test in the near future.

Several strategies have been reported to improve ergonomics in the OR and reduce the negative impact on surgeons; increased awareness of these amongst surgeons is undoubtedly a good strategy to avoid long term health sequelae [34]. This study found a significant correlation between operations greater than 3 h and increased MSK discomfort. It is of course unrealistic to try to modify the length of such procedures but it has been shown that taking microbreaks may be of benefit for the scrubbed team and that this does not impact on length of surgery [9, 35, 36]. Other strategies to mitigate discomfort/pain which potentially leads to MSK injury or reduced career longevity include postural awareness using observation or wearable sensors, changing surgeons’ postures by performing some procedures standing and others seated [37–39], performing a variety of procedures to change the load periodically for the surgeon, placement of trocars or other means to optimize surgical approach, and using other interventions such as armrests or fixtures for retraction.

Surgery is a physical profession that requires stamina and steps to improve the general fitness of surgeons, akin to the approach taken with airline pilots, could also be an effective prevention strategy for MSK injury [36, 40].

## Conclusions

This study indicates that MSK morbidity is a significant problem amongst EAES surgeons and is related to burn out and concerns over career longevity. The findings are consistent with similar studies and highlight the need for greater awareness, education and research into surgical ergonomics.
